# Topiramate Impairs Spermatogenesis, Sperm Parameters, and Epididymal Duct Contractility in Rats

**DOI:** 10.1021/acsptsci.6c00273

**Published:** 2026-08-15

**Authors:** Maele Oliveira de Sena, Maria Santana da Silva, Yanka do Vale Carvalho, Herick Jose Cunha Chaves, Maria Letícia da Costa, Bruna da Silva Figueredo, Jackson Nazareno Gomes de Lima, Renata Souza e Silva, Expedito Silva do Nascimento Junior, Anna Karynna Alves de Alencar Rocha, Elaine Cristina Gavioli, Edilson Dantas da Silva Junior

**Affiliations:** † Department of Biophysics and Pharmacology, 28123 Federal University of Rio Grande Do Norte, Natal, 59072-970 Rio Grande do Norte, Brazil; ‡ Department of Morphology, Federal University of Rio Grande do Norte, Natal 59072-970 Rio Grande do Norte, Brazil; § Maurício de Nassau University Center, Natal 59080-400 Rio Grande do Norte, Brazil

**Keywords:** topiramate, spermatogenesis, epididymis, sperm quantity and quality, epididymal contractility

## Abstract

Topiramate is a widely prescribed antiepileptic drug with a multimodal mechanism of action. However, its effects on male reproductive function remain poorly characterized. This study was conducted to investigate the impact of this drug on testicular and epididymal function in adult male Wistar rats. Animals were treated orally for 21 days with topiramate at 40 or 160 mg/kg. Serum testosterone levels, testicular histomorphology, spermatogenic indices, sperm parameters, and epididymal duct contractility were subsequently evaluated. The treatment did not alter serum testosterone levels at either dose. However, both doses reduced Sertoli cell and round spermatid numbers, increased primary spermatocyte counts, and decreased meiotic and overall spermatogenic yields, whereas only the higher dose (160 mg/kg) reduced Sertoli cell efficiency and daily sperm production. The lower dose (40 mg/kg) increased sperm morphological abnormalities, whereas the higher dose reduced total and progressive sperm motility and induced both head and tail defects. Additionally, treatment with topiramate at 160 mg/kg prolonged sperm transit time in the cauda epididymis and reduced contractile responses of the distal cauda epididymal duct to KCl, noradrenaline, and carbachol, suggesting impaired epididymal transport and potential ejaculatory dysfunction. Altogether, these findings demonstrate that topiramate adversely affects male reproductive function. Given its widespread use in individuals of reproductive age, these results underscore the need for translational studies in humans to assess potential reproductive risks associated with this therapy.

## Graphical abstract

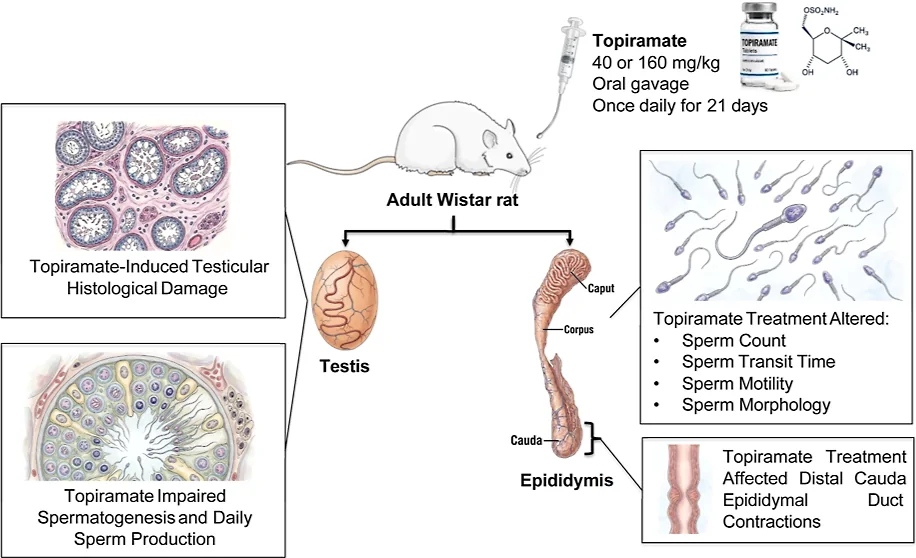

Male infertility affects approximately 7% of the global male population and is increasingly recognized as a multifactorial condition influenced by lifestyle, environmental, and pharmacological factors.^1^ Among pharmacological contributors, central nervous system (CNS)-acting drugs, particularly antiepileptic agents, have gained attention for disrupting endocrine signaling, spermatogenesis, and sperm quality.^2^

Topiramate, a second-generation antiepileptic drug, exhibits a multimodal mechanism of action that includes blockade of voltage-gated sodium and calcium channels, potentiation of GABA_A_-mediated inhibitory neurotransmission, antagonism of AMPA/kainate glutamate receptors, and inhibition of carbonic anhydrase (CA) isoforms II and IV.^3,4^ Beyond epilepsy, it is widely prescribed for migraine prophylaxis, neuropathic pain, bipolar disorder, and obesity, resulting in extensive use, including among individuals of reproductive age.^5^ Despite this, its impact on male reproductive function remains poorly defined.

Other antiepileptic drugs, such as carbamazepine, lamotrigine, oxcarbazepine, and valproate, are known to cause male reproductive adverse effects through both endocrine disruption and direct gonadal actions, including altered Sertoli cell function and germ cell dynamics, ultimately compromising spermatogenesis.^2,6^ For topiramate, however, evidence remains scarce and largely limited to a few experimental studies reporting reduced testosterone levels and/or impaired sperm production in rats.^7,8^

Spermatogenesis is a tightly regulated process that depends on the structural integrity of the seminiferous epithelium, coordinated Sertoli cell support, and precise hormonal control.^9,10^ As such, it is highly susceptible to pharmacological interference, particularly from agents that disrupt endocrine signaling or directly target testicular cell populations. Impairment of germ cell proliferation or differentiation ultimately reduces sperm production and compromises sperm quality.^2,11,12^

Beyond testicular effects, male reproductive capacity also depends on post-testicular events in the epididymis, where spermatozoa undergo maturation, transport, and storage.^2,12^ These processes are tightly regulated by coordinated contractile activity of the epididymal duct, which governs sperm transit and is essential for acquiring motility and fertilizing capacity.^13^ This contractile function is Ca^2+^-dependent and modulated by adrenergic and cholinergic neurotransmitters, as well as local mediators such as prostaglandins and endothelin.^13^ Disruption of these regulatory mechanisms alters sperm transit dynamics, leading to defective maturation, abnormal storage, and reduced fertilizing potential.^12,13^ Accordingly, pharmacological agents that target ion channels, autonomic neurotransmission, or smooth muscle signaling can impair epididymal motility and negatively affect sperm parameters.^14–18^ Given its multimodal pharmacological profile, we hypothesized that topiramate may interfere with both testicular and epididymal function. In particular, its effects on testosterone levels,^7^ Ca^2+^ handling,^19^ and autonomic signaling^20^ suggest a previously underexplored capacity to disrupt epididymal duct contractility, thereby altering sperm transport as well as sperm quantity and quality. Here, we systematically evaluated the effects of topiramate on male reproductive function in rats by integrating testicular histomorphological analysis, quantitative and qualitative sperm assessment, and functional pharmacological evaluation of epididymal duct contractility.

## Materials and Methods

### Animals

A total of 33 male Wistar rats (3–4 months old, weighing 400–600 g) were used. Animals were housed in the Animal Facility of the Department of Biophysics and Pharmacology (DBF) in polypropylene cages (four animals per cage) with ad libitum access to water and food (Purina, Labina), under a 12 h light–dark cycle (lights on from 6:00 a.m. to 6:00 p.m.) and controlled temperature (23 ± 1 °C). All animal experiments were performed following the protocols evaluated and approved by the Animal Ethics Committee of the Federal University of Rio Grande do Norte (Ethics Approval Number: 046/2024). Every effort was made to minimize animal distress and suffering.

### Treatment and Experimental Groups

Rats were randomly assigned to experimental groups and treated orally (by gavage) for 21 days with topiramate at 40 or 160 mg/kg. The control group received saline solution (0.9% NaCl) via the same route and volume. Doses were selected to correspond to clinically relevant human doses (minimum effective dose: 200–400 mg/day; up to a maximum recommended dose of 1600 mg/day in some cases). Using the interspecies dose conversion formula (animal equivalent dose = human equivalent dose × 6.2, correction factor for rats) described by Nair and Jacob,^21^ and assuming a 60 kg adult, the animal dose of 40 mg/kg corresponds to a human equivalent dose of 6.45 mg/kg (approximately 400 mg/day), whereas 160 mg/kg corresponds to 25.8 mg/kg (approximately 1500 mg/day).

Animals were weighed daily before drug administration. At the end of the 21 day period, they were euthanized by decapitation, and blood samples were collected for serum testosterone measurement. Reproductive organs (testes, epididymides, prostate, vas deferens, and seminal vesicles) were then excised, weighed, and processed for histopathological analysis, sperm parameter assessment, and isolated organ bath experiments, as described below. Both absolute and relative organ weights (normalized to body weight) were evaluated.

### Experimental Design and Allocation of Animals

The study was conducted in two sequential experimental sets. In the first set, 14 rats were used (7 controls and 7 receiving topiramate at 40 mg/kg). In the second set, an additional 14 rats were included (7 controls and 7 receiving topiramate at 160 mg/kg), resulting in a total of 28 animals. Animals were allocated to different experimental end points according to tissue availability and methodological requirements. Blood samples were collected from all animals. However, due to insufficient sample volume, serum testosterone analyses were performed in 12 control animals, 5 animals from the topiramate 40 mg/kg group, and 7 animals from the 160 mg/kg group.

For histological analysis and sperm evaluation, the same animals described above were used (*n* = 7 per topiramate group). Because 14 control animals were available (7 from each experimental set), a randomized subset of seven control animals was selected from the pooled control group, resulting in balanced groups (*n* = 7 per group). For in vivo contractility experiments, a subset of five animals per group was randomly selected from the animals described above and evaluated immediately after euthanasia because of the short posteuthanasia viability window required for functional assessment of the distal cauda epididymal duct. An additional cohort of 5 animals was used for in vitro contractility experiments.

### Determination of Serum Testosterone Levels

Blood samples were centrifuged to obtain serum, and testosterone concentrations were deter-mined using the VITROS MicroWell Testosterone immunoassay (Reagent Pack, catalog no. 1435205; Ortho Clinical Diagnostics, Raritan, NJ, USA) according to the manufacturer’s instructions on the VITROS 5600 Immunodiagnostic System. This competitive chemiluminescent immunoassay is compatible with the VITROS ECi/ECiQ, 3600, and XT 7600 Immunodiagnostic Systems. It has an analytical measurement range of 4.90–2160 ng/dL, with intra-assay coefficients of variation ranging from 1.1% to 3.6% and within-laboratory (interassay) coefficients of variation ranging from 3.1% to 5.1%, according to the manufacturer. This assay has previously been employed for serum testosterone determination in rats.^22^ Hormone quantification was performed to determine whether the alterations detected were associated with hormonal changes or with direct effects of topiramate on testicular cell populations or the epididymal duct.

### Testicular Histomorphological Analysis

Testes were excised and fixed in Bouin’s solution for 24 h, then embedded in paraffin. Sections (4 μm thick) were mounted on glass slides and stained with hematoxylin and eosin for histomorphological evaluation under a light microscope.

### Quantification of Seminiferous Tubule Cell Populations

Histological analyses were performed using a light microscope (Olympus BX40) coupled to an Image-Pro Express (v.6.0) image analysis system. Images of seminiferous tubules were captured to measure nuclear diameters of 10 cells from each evaluated cell type per testis. In addition, the nucleolar diameter of Sertoli cells was measured.

Three histological slides per testis (six per animal) were examined. Stage VII of the seminiferous epithelium cycle (SEC) was identified based on tubular morphology according to Ortavant et al.,^23^ considering changes in spermatid nuclear morphology, meiotic divisions, and spermatid position within the epithelium. Cell quantification was performed in ten cross sections of seminiferous tubules per animal, preferentially selecting those with a circular profile. Counted cell types included type A spermatogonia, primary spermatocytes (preleptotene/leptotene), round spermatids, and Sertoli cells (identified by their nucleoli). All analyses were performed blinded to experimental groups.

Cell counts were corrected using the eq 1 proposed by Abercrombie^24^ and later modified by Amann and Almquist^25^

1 $$corrected\;number=observed\;count\times \frac{section\;thickness}{section\;thickness+\sqrt{{\left(MD/2\right)}^{2}-{\left(MD/4\right)}^{2}}}$$

where MD is the mean nuclear diameter. This correction accounts for both nuclear diameter and section thickness. For Sertoli cells, nucleolar diameter was used instead of nuclear diameter due to high morphological variability and the difficulty of precisely defining nuclear boundaries.

### Spermatogenic Yield and Sertoli Cell Efficiency

These parameters were calculated from cell populations in ten cross sections of seminiferous tubules, as follows: (i) Mitotic yield (mitotic efficiency coefficient): ratio of primary spermatocytes at the preleptotene/leptotene stages to type A spermatogonia; (ii) Meiotic yield: ratio of round spermatids to primary spermatocytes at preleptotene/leptotene stages; (iii) Overall spermatogenic yield: ratio of round spermatids to type A spermatogonia; and (iv) Sertoli cell efficiency: ratio of round spermatids to Sertoli cells.

### Testicular Histometry

Ten cross sections of seminiferous tubules at stage VII of the seminiferous epithelium cycle were analyzed, preferentially those with a circular morphology, to measure tubular diameter and area, luminal area, and seminiferous epithelium height. The evaluator was blinded to group allocation throughout all analyses. Images were captured using a light microscope (Olympus BX40) equipped with a 40× objective and a digital camera. Histometric measurements were performed using ImageJ (version 1.54g; National Institutes of Health, Bethesda, MD, USA).

### Determination of Sperm Parameters

#### Daily Sperm Production, Sperm Transit Time, and Epididymal Sperm Concentration

Testes and epididymides from topiramate-treated and control animals were excised and processed to determine daily sperm production, sperm transit time, and epididymal sperm concentration. For daily sperm production, testes were decapsulated, weighed, and homogenized in 5 mL of 0.9% NaCl containing 0.5% Triton X-100, followed by sonication for 30 s. The homogenate was then diluted 10-fold, and an aliquot was transferred to a Neubauer chamber for counting stage 19 spermatids, which are homogenization-resistant, mature elongated spermatids present immediately before spermiation,^23,26^ in four fields per animal. Daily sperm production was calculated by dividing the total number of spermatids per testis by 6.1, corresponding to the number of days these spermatids remain in the seminiferous epithelium.^26^

Epididymides were divided into two regions (caput/corpus and cauda). Each region was homogenized, and sperm counts were determined as described for spermatids. Sperm transit time through the epididymis was calculated by dividing the number of spermatozoa in each segment by the corresponding daily sperm production.^27^

#### Sperm Motility Analysis

Epididymal fluid samples from treated and control animals were collected by making small incisions (<1 mm) in the cauda epididymis. Samples were resuspended in 1 mL of phosphate-buffered saline (PBS: 137 mM NaCl, 2.7 mM KCl, 10 mM Na_2_HPO_4_, 1 mM KH_2_PO_4_, pH 7.4) and analyzed for motility under a light microscope. A total of 200 spermatozoa were evaluated per sample to determine total, progressive, and nonprogressive motility (%).

#### Sperm Viability Analysis

Epididymal fluid samples were collected as described above, diluted 1:1 with 5% eosin Y solution, gently mixed, and a drop was placed on a microscope slide and covered with a coverslip for analysis. A total of 200 spermatozoa were examined and classified as live or dead based on membrane integrity. Viability was expressed as the percentage of live spermatozoa.

#### Sperm Morphology Assessment

Epididymal fluid samples from treated and control animals were obtained as described for motility analysis. An aliquot (20 μL) was placed on a glass slide, and a smear was prepared. A total of 200 spermatozoa were examined under a light microscope and classified as normal or abnormal. Morphologically abnormal spermatozoa were further categorized according to the type of defect, including head, midpiece, or tail abnormalities.

### Functional Experiments in Isolated Organ Bath

Segments (1.5 cm) of the distal cauda epididymal duct from topiramate-treated and control animals were isolated and mounted under an initial tension of 1.0 g in an organ bath containing a nutrient solution maintained at 32 °C with the following composition (mM): 138.0 NaCl, 5.7 KCl, 1.8 CaCl_2_, 0.36 NaH_2_PO_4_, 15 NaHCO_3_, and 5.5 glucose, prepared in distilled water, continuously aerated, and maintained at pH 7.35–7.40. One end of each tissue was fixed to a glass rod, and the other was connected to a force transducer coupled to a physiograph for recording isometric contractions. Contractions were induced by cumulative addition of increasing concentrations of agonists (noradrenaline or carbachol) to generate cumulative concentration–effect curves (CCECs), or by a single concentration of KCl (80 mM). In separate experiments, the in vitro effects of topiramate (0.1, 1, and 10 μM) were evaluated on CCECs to agonists in epididymal ducts from untreated rats. From the CCECs, pharmacological parameters were determined, including maximal effect (*E*_max_, indicative of efficacy) and the negative logarithm of the concentration producing 50% of the maximal effect (−log EC_50_, indicative of potency), allowing comparisons among experimental groups. Detailed methodologies have been previously described in studies from our group.^16–18^

### Drugs and Reagents

All drugs were obtained from Sigma Chemical Co. (St. Louis, MO, USA), whereas all other reagents were purchased from Dinâmica (Brazil).

### Data Analysis

Data are presented as mean ± standard error of the mean (SEM). The normality of the data distribution was assessed using the Shapiro–Wilk test. As all data sets showed a normal distribution, comparisons among three or more groups were performed using one-way analysis of variance (ANOVA), followed by Tukey’s test. A significance level of 5% (*p* < 0.05) was adopted for all analyses. Statistical analyses were performed using GraphPad Prism 9 (GraphPad Software, San Diego, CA, USA).

## Results

### Body and Reproductive Organ Weights

Treatment with topiramate (40 and 160 mg/kg) for 21 days significantly reduced body weight compared to controls. While control animals gained slight weight, treated animals lost weight, as indicated by a negative body weight variation (Table 1).

**Table 1:** Body Weight and Reproductive Organ Weights of Animals Exposed to Oral (Gavage) treatment with topiramate (40 or 160 mg/kg) or Vehicle (Control) for 21 days^a^

Parameters	Control (*n* = 14)	Topiramate 40 mg/kg (*n* = 7)	Topiramate 160 mg/kg (*n* = 7)
Body Weight			
Initial (g)	555.3 ± 10.6	531.3 ± 18.6	480.4 ± 12.3
Final (g)	560.5 ± 13.3	503.4 ± 13.3	462.3 ± 10.3
Weight variation (%)	0.8 ± 1.0	–5.0 ± 1.5*	–3.6 ± 1.8*
Absolute Organ Weight (g)			
Testis	1.81 ± 0.07	1.77 ± 0.06	1.77 ± 0.04
Empty seminal vesicle	0.53 ± 0.02	0.42 ± 0.03	0.53 ± 0.03
Vas deferens	0.13 ± 0.01	0.09 ± 0.01	0.12 ± 0.01
Prostate	0.77 ± 0.09	0.36 ± 0.05*	1.11 ± 0.03*^,#^
Epididymis	0.71 ± 0.03	0.74 ± 0.02	0.73 ± 0.02
Relative Organ Weight (g tissue/100 g body weight)			
Testis	0.32 ± 0.01	0.35 ± 0.009	0.38 ± 0.01*
Empty seminal vesicle	0.09 ± 0.01	0.08 ± 0.01	0.11 ± 0.01
Vas deferens	0.02 ± 0.001	0.02 ± 0.002	0.02 ± 0.003
Prostate	0.14 ± 0.02	0.07 ± 0.01*	0.24 ± 0.01*^,#^
Epididymis	0.12 ± 0.006	0.14 ± 0.001*	0.15 ± 0.004*

a Values are expressed as mean ± SEM *n*, sample size. Body weight variation (%) was calculated as [(final body weight – initial body weight)/initial body weight] × 100. Data were analyzed by one-way ANOVA followed by Tukey’s test. *p < 0.05 vs control. ^#^ *p* < 0.05 vs topiramate 40 mg/kg.

Absolute and relative reproductive organ weights were also evaluated. The 40 mg/kg dose significantly reduced prostate weight, whereas the 160 mg/kg dose increased it compared to controls or 40 mg/kg group. No significant differences were observed in absolute weights of testes, seminal vesicles, vas deferens, or epididymis (Table 1). Regarding relative weights, the 40 mg/kg dose decreased prostate weight but increased relative epididymis weight, while the 160 mg/kg dose increased relative weights of testes, prostate, and epididymis (Table 1).

### Serum Testosterone Levels in Animals Treated with Topiramate

Treatment with topiramate at 40 or 160 mg/kg for 21 days did not significantly alter serum testosterone levels compared to the control group (Figure 1).

**Figure 1:**
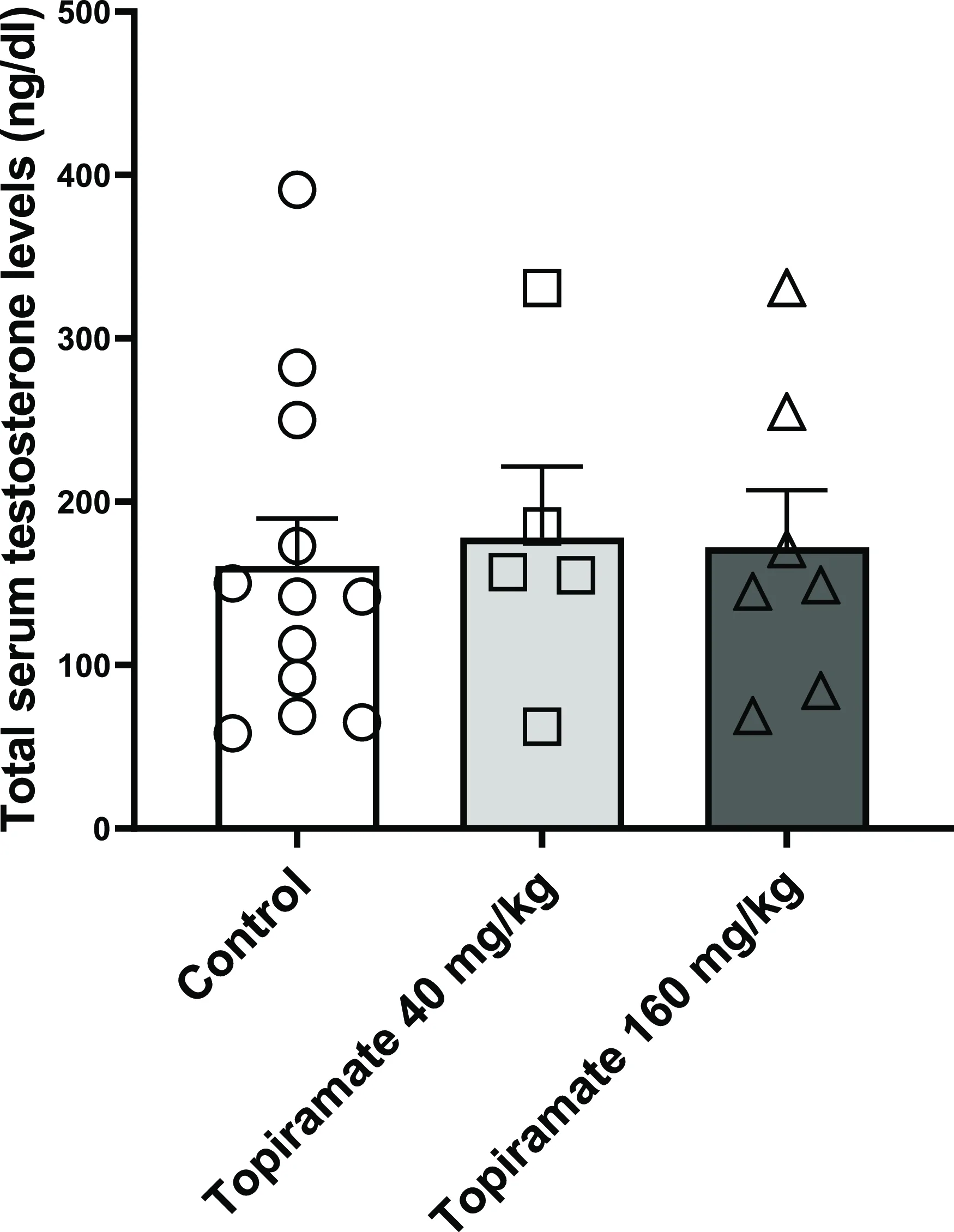
Total serum testosterone levels in control animals and those treated with topiramate at 40 or 160 mg/kg. Each bar represents the mean ± SEM of 12 samples for the control group, 5 samples for the 40 mg/kg group, and 7 samples for the 160 mg/kg group. Dots represent individual values. Data were analyzed by one-way ANOVA followed by Tukey’s test.

### Effects of Topiramate Treatment on Testicular Histomorphology, Seminiferous Epithelium Cell Populations, and Histometry

Histomorphological analysis of the testes revealed normal morphology in control animals, with a well-organized and robust seminiferous epithelium (Figure 2A). In contrast, animals treated with topiramate at 40 mg/kg exhibited reduced seminiferous epithelium thickness and increased luminal space (Figure 2B). More pronounced alterations were observed in the 160 mg/kg group, including marked disruption of seminiferous tubule architecture, characterized by epithelial atrophy, as evidenced by a thinner seminiferous epithelium than in controls, along with pronounced luminal dilation and a relative depletion of spermatozoa within the lumen (Figure 2C).

**Figure 2:**
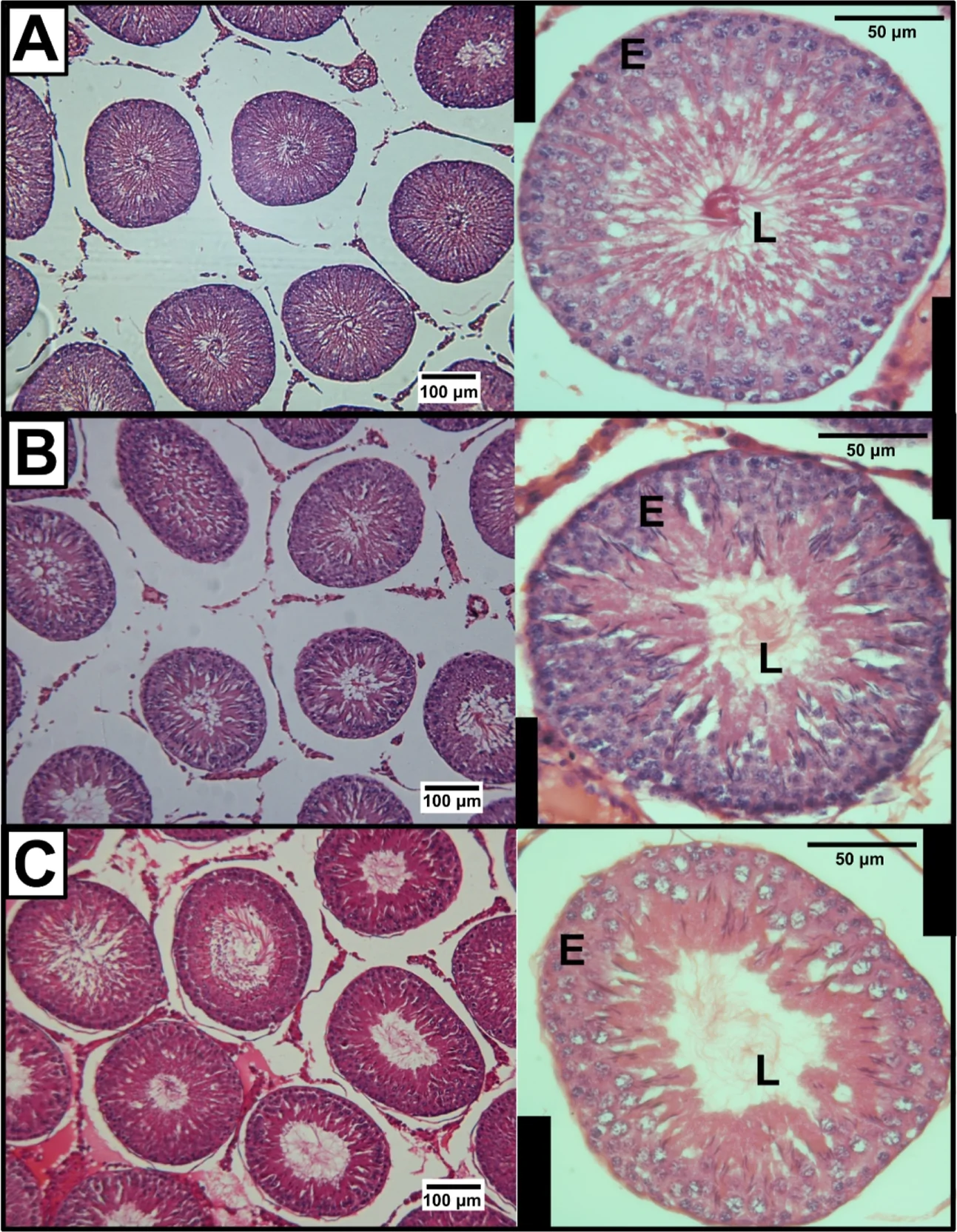
Histological sections showing seminiferous tubules in the testes of control animals (panel (A); right, 10×; left, 40×), animals treated with topiramate at 40 mg/kg (panel (B); right, 10×; left, 40×), and animals treated with topiramate at 160 mg/kg (panel (C); right, 10×; left, 40×) for 21 days. E, seminiferous epithelium; L, lumen.

Topiramate treatment for 21 days significantly altered seminiferous epithelium cell populations. Treatment with 40 and 160 mg/kg reduced Sertoli cell numbers by approximately 33% and 26%, respectively. The same doses increased primary spermatocyte (PL/L) counts by approximately 33% and 23%, while reducing round spermatid numbers by approximately 38% and 47%, respectively, compared with controls. No significant changes were observed in type A spermatogonia counts at either dose (Table 2).

**Table 2:** Cell Counts per Seminiferous Tubule Cross-Section in Rats Exposed to In Vivo Treatment with topiramate or Vehicle (Control) for 21 Days^a^

Cells	Control (*n* = 7)	Topiramate 40 mg/kg (*n* = 7)	Topiramate 160 mg/kg (*n* = 7)
Sertoli cells	15.8 ± 0.49	10.6 ± 0.80*	11.7 ± 0.48*
Type A spermatogonia	11.5 ± 0.83	13.1 ± 0.57	13.2 ± 0.30
Primary spermatocytes (PL/L)	33.1 ± 0.86	43.9 ± 2.18*	40.7 ± 1.47*
Round spermatids	95.0 ± 5.35	58.5 ± 5.05*	50.8 ± 3.98*

a Values are expressed as mean ± SEM *n*, sample size. PL/L = preleptotene and leptotene spermatocytes. Data were analyzed by one-way ANOVA followed by Tukey’s test. *p < 0.05 vs control. *Note:* Cell counts were corrected using the Abercrombie method,^24^ modified by Amann and Almquist.^25^

Topiramate treatment significantly affected spermatogenic efficiency indices. Compared with controls, meiotic yield decreased by approximately 52% and 55%, and spermatogenic yield by approximately 38% and 54% following treatment with 40 and 160 mg/kg, respectively. Sertoli cell efficiency was reduced by about 27% at the highest dose, whereas mitotic yield remained unchanged (Table 3).

**Table 3:** Spermatogenic Indices and Sertoli Cell Efficiency in Rats Exposed to In Vivo Treatment with topiramate or Vehicle (Control) for 21 Days^a^

Parameters	Control (*n* = 7)	Topiramate 40 mg/kg (*n* = 7)	Topiramate 160 mg/kg (*n* = 7)
Mitotic yield	3.0 ± 0.16	3.3 ± 0.14	3.1 ± 0.08
Meiotic yield	2.9 ± 0.18	1.4 ± 0.15*	1.3 ± 0.12*
Spermatogenic yield	8.4 ± 0.62	5.2 ± 0.52*	3.9 ± 0.35*
Sertoli cell efficiency	6.0 ± 0.28	5.50 ± 0.35	4.4 ± 0.44*

a Values are expressed as mean ± SEM *n*, sample size. Data were analyzed by one-way ANOVA followed by Tukey’s test. *p < 0.05 vs control.

Histometric analysis revealed that both topiramate doses significantly increased luminal area and reduced seminiferous epithelium height (Table 4).

**Table 4:** Seminiferous Tubular Diameter (STD), Seminiferous Tubule Area (STA), Luminal Area (LA), and Seminiferous Epithelium Height (SEH) in Rats Exposed to In Vivo Treatment with Topiramate or Vehicle (Control) for 21 Days^a^

Parameters	Control (*n* = 7)	Topiramate 40 mg/kg (*n* = 7)	Topiramate 160 mg/kg (*n* = 7)
Seminiferous tubular diameter (μm)	264.3 ± 5.4	256.7 ± 3.5	258.5 ± 5.4
Seminiferous tubule area (×10^3^ μm^2^)	56.7 ± 1.3	52.7 ± 1.0	55.5 ± 1.5
Luminal area (×10^3^ μm^2^)	3.0 ± 0.4	4.3 ± 0.4*	4.9 ± 0.4*
Seminiferous epithelium height (μm)	96.8 ± 2.1	90.1 ± 1.3*	89.2 ± 1.6*

a Values are expressed as mean ± SEM *n*, sample size. Data were analyzed by one-way ANOVA followed by Tukey’s test. *p < 0.05 vs control.

### Effects of Topiramate Treatment on Quantitative Sperm Parameters

The 40 mg/kg dose increased sperm counts in the cauda epididymis and prolonged transit time in this region, without significantly affecting spermatid number, daily sperm production, or sperm counts and transit time in the caput/corpus (Table 5).

**Table 5:** Quantitative Sperm Parameters in Rats Exposed to Oral (Gavage) Treatment with Topiramate (40 or 160 mg/kg) or Vehicle (Control) for 21 Days^a^

Sperm parameters	Control (*n* = 7)	Topiramate 40 mg/kg (*n* = 7)	Topiramate 160 mg/kg (*n* = 7)
Testis			
Spermatids number (×10^6^/testis)	288.8 ± 11.8	237.3 ± 25.3	184.6 ± 20.0*
Spermatids number (×10^6^/g testis)	191.5 ± 5.5	171.0 ± 10.0	125.9 ± 15.4*^,#^
Daily sperm production (×10^6^/testis/day)	47.3 ± 1.9	38.9 ± 4.1	30.2 ± 3.3*
Daily sperm production (×10^6^/g testis/day)	31.4 ± 0.9	28.0 ± 1.6	20.6 ± 2.5*^,#^
Caput/Corpus Epididymis			
Sperm number (×10^6^/organ)	154.8 ± 7.5	149.3 ± 17.0	98.8 ± 8.8*^,#^
Sperm number (×10^6^/g organ)	364.0 ± 26.8	401.8 ± 51.6	297.6 ± 19.9
Transit time (days)	3.3 ± 0.2	4.3 ± 0.9	3.7 ± 0.7
Cauda Epididymis			
Sperm number (×10^6^/organ)	236.8 ± 19.7	307.6 ± 38.5	214.4 ± 11.2^#^
Sperm number (×10^6^/g organ)	1001.0 ± 60.3	1278.0 ± 108.8*	949.7 ± 56.3^#^
Transit time (days)	4.0 ± 0.3	8.2 ± 1.3*	7.8 ± 1.2*

a Values are expressed as mean ± SEM *n*, sample size. Data were analyzed by one-way ANOVA followed by Tukey’s test. *p < 0.05 vs control. ^#^ *p* < 0.05 vs topiramate 40 mg/kg.

The 160 mg/kg dose reduced testicular spermatid number and daily sperm production compared with the control group. When normalized to testis weight, these reductions were also significant compared with the 40 mg/kg group. Sperm counts in the caput/corpus epididymis were likewise reduced compared with both the control and 40 mg/kg groups. However, this difference was not observed when sperm counts were normalized to organ weight. Sperm counts in the cauda epididymis remained unchanged compared with the control group, likely due to prolonged transit time in this segment (Table 5).

### Effects of Topiramate Treatment on Qualitative Sperm Parameters

Sperm motility, viability, and morphology were evaluated in samples collected from the cauda epididymis of control rats and those treated with topiramate at 40 mg/kg or 160 mg/kg for 21 days. The 40 mg/kg dose reduced the proportion of morphologically normal spermatozoa, accompanied by a significant increase in morphological abnormalities, mainly involving the sperm tail (Figure 3C). At this dose, no significant changes were observed in sperm motility or viability (Figure 3A,B).

**Figure 3:**
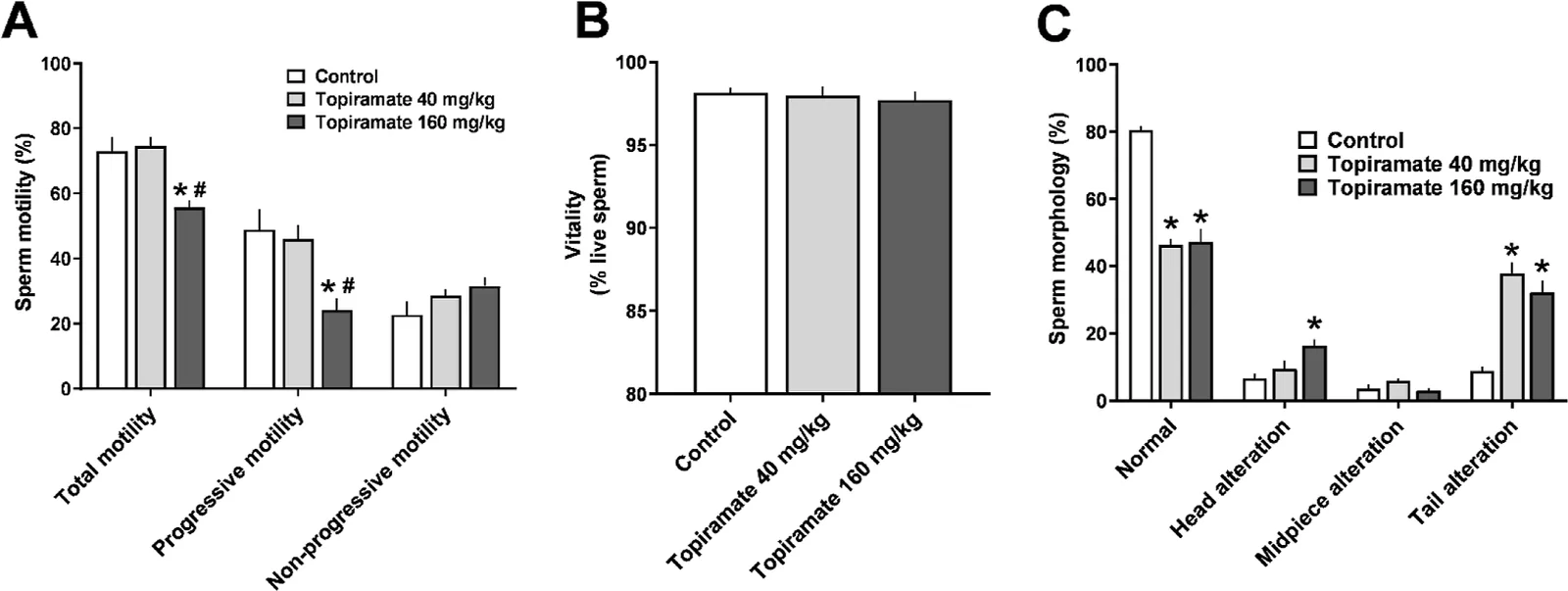
Evaluation of sperm motility (A), viability (B), and morphology (C) in samples collected from the distal cauda epididymis of rats treated orally (gavage) with topiramate (40 or 160 mg/kg) or vehicle (control) for 21 days. Each bar represents the mean ± SEM of 7 samples. Data were analyzed by one-way ANOVA followed by Tukey’s test. *p < 0.05 vs control. ^#^ *p* < 0.05 vs topiramate 40 mg/kg.

Conversely, the 160 mg/kg dose significantly reduced total and progressive sperm motility compared with the control and 40 mg/kg group. Although viability remained unchanged (Figure 3B), a significant reduction in morphologically normal spermatozoa was observed, associated with increased abnormalities affecting both the sperm head and tail (Figure 3C).

### Effects of Topiramate on Contractile Responses of the Isolated Distal Cauda Epididymal Duct in Rats

In vitro preincubation for 30 min with topiramate (0.1–10 μM) did not significantly alter contractile responses of the epididymal duct induced by cumulative addition of noradrenaline (adrenergic agonist) or carbachol (cholinergic agonist) (Figure 4A,B and Table 6).

**Figure 4:**
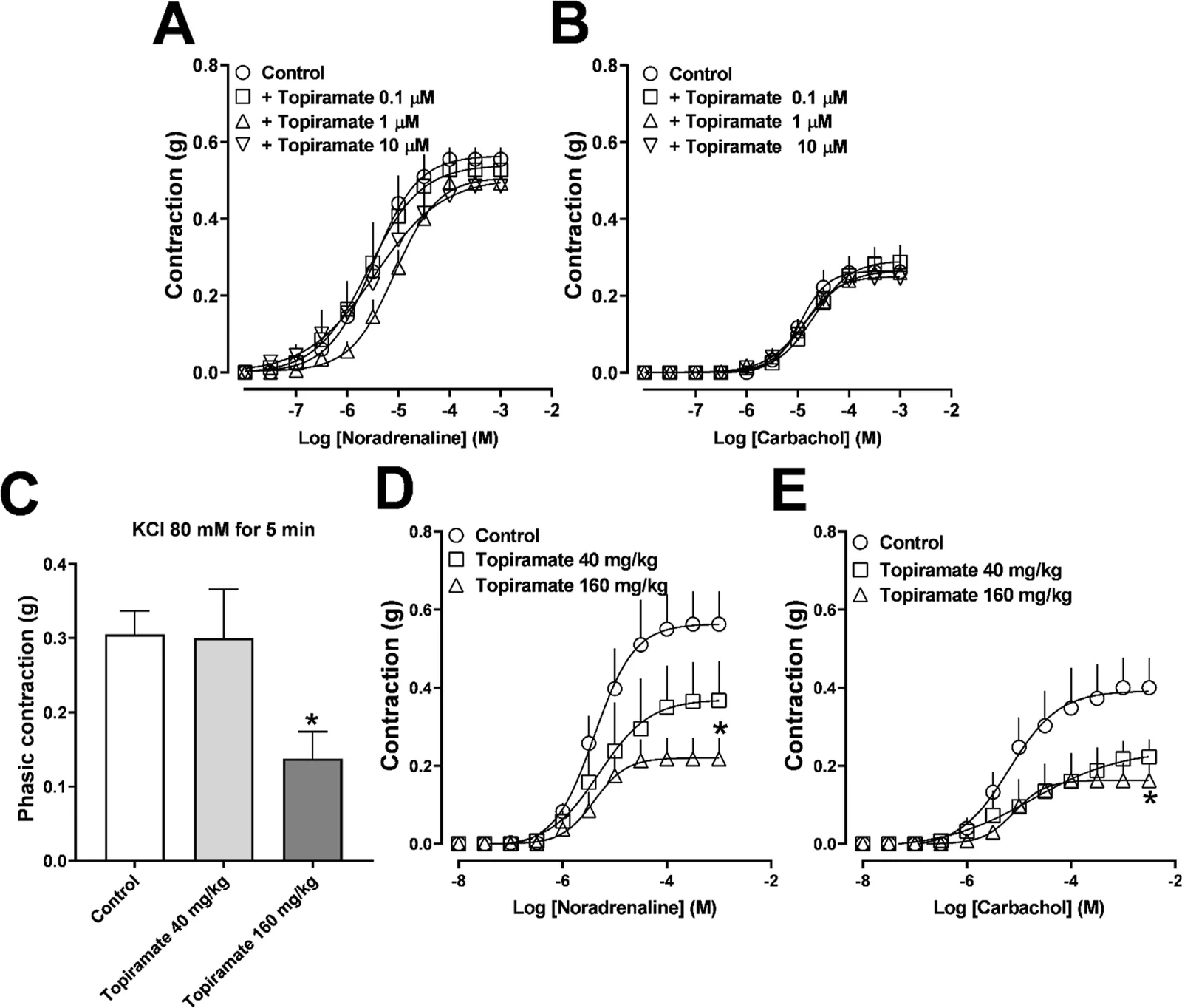
In vitro effects of topiramate (0.1–10 μM) on contractile responses of isolated distal cauda epididymal duct segments assessed by cumulative concentration–response curves to noradrenaline (A) and carbachol (B). Contractile responses of distal cauda epididymal duct segments from rats treated in vivo with vehicle (control) or topiramate (40 or 160 mg/kg, 21 days) are shown in panels (C–E). (C) Contractions induced by a single concentration of KCl (80 mM, 5 min). Concentration–response curves to noradrenaline (D) and carbachol (E). Each bar (C) and each point (A,B,D,E) represents the mean ± SEM of 5 independent experiments. Data were analyzed by one-way ANOVA followed by Tukey’s test. *p < 0.05 vs control.

**Table 6:** Maximum Effect (*E*_max_) and Potency (pEC_50_) of Noradrenaline and Carbachol in the Absence (Control) or in the Presence of Topiramate (0.1–10 μM, In Vitro), as well as after In Vivo Treatment with Topiramate (40 or 160 mg/kg) or Vehicle (Control) for 21 Days^a^

	*E*_max_ (g)	pEC_50_
In Vitro		
Noradrenaline		
Control (*n* = 5)	0.55 ± 0.03	5.5 ± 0.22
+ topiramate 0.1 μM (*n* = 5)	0.53 ± 0.05	5.5 ± 0.27
+ topiramate 1 μM (*n* = 5)	0.49 ± 0.04	5.1 ± 0.14
+ topiramate 10 μM (*n* = 5)	0.48 ± 0.06	5.5 ± 0.36
Carbachol		
Control (*n* = 5)	0.26 ± 0.04	4.9 ± 0.10
+ topiramate 0.1 μM (*n* = 5)	0.29 ± 0.04	4.7 ± 0.13
+ topiramate 1 μM (*n* = 5)	0.26 ± 0.05	4.9 ± 0.15
+ topiramate 10 μM (*n* = 5)	0.24 ± 0.06	4.9 ± 0.06
In Vivo		
Noradrenaline		
Control (*n* = 5)	0.56 ± 0.08	5.3 ± 0.24
Topiramate 40 mg/kg (*n* = 5)	0.37 ± 0.10	4.9 ± 0.29
Topiramate 160 mg/kg (*n* = 5)	0.22 ± 0.05*	5.3 ± 0.16
Carbachol		
Control (*n* = 5)	0.40 ± 0.07	4.8 ± 0.32
Topiramate 40 mg/kg (*n* = 5)	0.22 ± 0.04	4.5 ± 0.45
Topiramate 160 mg/kg (*n* = 5)	0.16 ± 0.04*	5.1 ± 0.11

a Values are expressed as mean ± SEM of 5 independent experiments performed using tissues from different animals. *E*_max_ is expressed in grams of contraction. Data were analyzed by one-way ANOVA followed by Tukey’s test. *p < 0.05 vs control.

Treatment with topiramate at 40 mg/kg for 21 days did not significantly affect contractions of distal cauda epididymal duct segments induced by KCl, noradrenaline, or carbachol (Figure 4C–E and Table 6). In contrast, in vivo the 160 mg/kg dose significantly reduced contractions induced by the depolarizing agent KCl (80 mM, 5 min) (Figure 4C), as well as by noradrenaline and carbachol, as evidenced by a significant decrease in *E*_max_ values for both agonists (Figure 4D,E and Table 6).

## Discussion

In humans, most studies on topiramate and reproduction have focused on teratogenic outcomes associated with gestational exposure, including increased risks of orofacial malformations and low birth weight.^28^ Direct evidence on its impact on male reproductive function, however, remains limited to a small number of experimental studies, reinforcing the relevance of animal models to characterize these effects.

In the present study, body weight reduction in topiramate-treated animals likely reflects its anorexigenic and weight-reducing properties.^7,29^ Changes in both absolute and relative weights of accessory sex organs are established markers of reproductive toxicity,^30,31^ although relative organ weights may be influenced by treatment-induced variations in body mass and should therefore be interpreted with caution. Here, treatment with topiramate at 40 mg/kg reduced both absolute and relative prostate weights, whereas the 160 mg/kg dose increased prostate weight in both absolute and relative terms, as well as relative testis weight. Relative epididymis weight was increased at both doses. Importantly, interpretation of these findings should consider the confounding effect of reduced body weight, which may contribute to apparent increases in relative organ weights.

In contrast, Otoom et al.^7^ reported generalized reductions in reproductive organ weights in rats treated with 100 mg/kg topiramate for 60 days, an effect largely associated with decreased testosterone levels. These discrepancies may be attributed to methodological differences, including treatment duration (60 vs 21 days) and rat strain (Sprague–Dawley vs Wistar), which are known to exhibit distinct endocrine and metabolic responses.^32,33^ Notably, serum testosterone levels remained unchanged in the present study. However, serum testosterone may not fully reflect intratesticular androgen concentrations or local androgen signaling. Therefore, the observed alterations in reproductive organ weights may involve mechanisms beyond changes in circulating testosterone, including tissue-specific sensitivity and/or indirect metabolic effects.

Testosterone, produced by Leydig cells, is essential for the maintenance of spermatogenesis, particularly during meiosis.^34^ Accordingly, drugs that reduce circulating testosterone levels tend to impair gamete production. Compounds such as fluoxetine, sertraline, acetylsalicylic acid, and dipyrone have been associated with decreased testosterone levels and reduced daily sperm production.^16,17^ Although some studies have suggested that topiramate may also reduce serum testosterone levels,^7^ no significant changes were observed in the present study after 21 days of treatment at either dose. Similarly, previous studies have reported that serum testosterone levels were not significantly affected by topiramate treatment in either humans^35^ or rats.^36^ Nevertheless, impairments in spermatogenesis were detected after topiramate treatment. These findings suggest that the drug may also exert direct effects on the seminiferous epithelium and highlight the value of histomorphological analyses in elucidating the underlying mechanisms.

Histological findings revealed that 21 days of topiramate treatment significantly altered spermatogenic dynamics. Both doses reduced Sertoli cell and round spermatid numbers while increasing primary spermatocyte counts, whereas only the 160 mg/kg dose reduced Sertoli cell efficiency. The reduction in Sertoli cell number and/or efficiency suggests compromised structural and metabolic support for normal spermatogenic progression, as these cells are pivotal for spermatogenic capacity.^37,38^ The accumulation of primary spermatocytes may indicate impaired progression through meiosis, possibly reflecting inadequate Sertoli cell support and/or reduced intratesticular testosterone.^39,40^ Accordingly, the marked reduction in round spermatids indicates impaired spermiogenesis and reduced sperm production potential. These findings are consistent with previous reports of decreased sperm count, reduced motility, and increased morphological abnormalities following topiramate exposure.^7^ The preservation of type A spermatogonia indicates that the basal germ cell compartment remained unaffected, suggesting that topiramate does not induce generalized cytotoxicity but primarily disrupts Sertoli cell function and later stages of germ cell differentiation.

These histological alterations were further supported by changes in spermatogenic efficiency indices. Reduced meiotic yield at both doses indicates impaired progression through meiosis, a critical step for haploid cell generation and sustained sperm production.^39,41^ Consistent with this finding, overall spermatogenic yield was markedly reduced, whereas Sertoli cell efficiency declined only at the higher dose.^38,39^ Together, these findings suggest that impaired Sertoli cell function may contribute to topiramate-induced spermatogenic dysfunction.

It should be noted that the present study evaluated the effects of topiramate after 21 days of treatment, which represents only an early period of exposure relative to the duration of complete spermatogenesis in rats (≈54 days)^42^ Therefore, the observed histological and functional alterations should be interpreted with caution and as evidence of early impairment of spermatogenic progression. Nevertheless, experimental models of male reproductive toxicity support the use of short-term exposure paradigms to detect early testicular injury. For instance, cadmium chloride induces rapid seminiferous epithelium injury, with early germ cell degeneration detectable within 7 days.^43^ Likewise, doxorubicin and cyclophosphamide cause time-dependent testicular damage, starting with early spermatocyte apoptosis at 7 days and progressing to germ cell loss at 21 days.^44^ Overall, these models demonstrate that early spermatogenic alterations can be detected within subacute exposure windows without requiring a full spermatogenic cycle. However, future studies employing longer treatment periods encompassing a complete spermatogenic cycle are warranted to determine the full extent of topiramate-induced reproductive effects.

Topiramate treatment was also associated with structural changes in the seminiferous tubules. Treated animals exhibited disruption of seminiferous epithelium integrity, with more severe alterations at 160 mg/kg. Increased luminal area, epithelial disorganization, and reduced epithelial height indicate germ cell loss, impaired cell–cell adhesion, and compromised structural support, all characteristic features of testicular toxicity.^45,46^

Reductions in epididymal sperm counts can be associated with altered spermatogenesis or impaired epididymal duct motility.^16,47^ In the 160 mg/kg group, decreased sperm numbers in the caput/corpus epididymis may reflect reduced sperm production. However, unchanged cauda sperm counts could reflect prolonged transit time in this segment, promoting sperm accumulation despite reduced production. Indeed, chemical denervation studies have shown that reduced duct motility prolongs transit time and increases local sperm concentration.^27^ Similarly, the increased sperm counts detected in the cauda epididymis of animals treated with topiramate at 40 mg/kg are consistent with delayed transit in this segment. Although the underlying mechanisms remain unclear, topiramate has been reported to modulate calcium channel activity,^19^ potentially altering spontaneous epididymal contractility. Because spontaneous contractions are essential for sperm transport throughout the epididymis,^13,14^ such alterations could impair sperm transit. However, this mechanism was not directly investigated in our study.

Topiramate treatment also affected sperm quality. The 40 mg/kg dose reduced the proportion of morphologically normal spermatozoa, whereas the 160 mg/kg dose impaired both sperm motility and morphology. In contrast, sperm viability remained unaffected at both doses. The preservation of sperm viability, despite reduced motility and increased morphological abnormalities, suggests that topiramate did not cause extensive plasma membrane damage to mature spermatozoa. Instead, these findings are more consistent with alterations acquired during spermatogenesis and/or epididymal maturation, resulting in the production of viable but structurally and functionally compromised spermatozoa.^48,49^

Beyond transit time, epididymal luminal fluid composition plays a key role in sperm maturation by providing ions, proteins, and epididymosomes that regulate sperm membrane remodeling and the acquisition of motility and fertilizing capacity.^50^ Topiramate is known to inhibit carbonic anhydrase isoforms, including CA II and CA IV,^51^ which are expressed in both the epididymal epithelium and spermatozoa, where they play a fundamental role in sperm maturation.^52,53^ In fact, knockout models for these isoforms exhibit reduced sperm motility.^53^ Because carbonic anhydrase activity was not directly evaluated in the present study, any contribution of topiramate-induced inhibition of this enzyme should be considered a plausible but unverified mechanism.

In the distal cauda epididymis, the duct functions primarily as a sperm storage site and exhibits minimal spontaneous contractile activity. During the emission phase of ejaculation, contractions of this segment, mediated by autonomic neurotransmitters, particularly noradrenaline acting on α_1A_-adrenoceptors and acetylcholine acting on M_3_ receptors, facilitate sperm transport from the epididymis to the vas deferens.^14,54,55^ Treatment with topiramate at 160 mg/kg reduced contractile responses to KCl, noradrenaline, and carbachol, indicating a nonspecific inhibitory effect that may impair ejaculatory function. Previous studies have suggested that topiramate may attenuate erectile and ejaculatory responses through mechanisms involving GABA_*A*_ receptor activation,^56^ leading to suppression of autonomic preganglionic pathways and spinal motor neurons involved in excitatory cholinergic, dopaminergic, and serotonergic signaling that regulate genital function.^57^ However, because acute in vitro exposure to topiramate did not alter contractions in isolated distal cauda epididymal ducts, the reduced contractility observed after in vivo treatment is unlikely to result solely from a direct pharmacological action on epididymal smooth muscle. Instead, it may reflect indirect systemic effects and/or treatment-induced structural or functional alterations of the epididymal duct. These mechanisms were not directly investigated in this study and should be regarded as plausible but unconfirmed explanations requiring further molecular and histological investigation.

## Conclusion

Our findings demonstrate that topiramate, mainly at the higher dose (160 mg/kg), induces significant alterations in male reproductive parameters in rats, including impaired spermatogenesis, reduced sperm motility, altered morphology, delayed epididymal sperm transit time, and decreased epididymal duct contractility. Collectively, these results indicate a potential adverse impact on male reproductive function. Beyond fertility itself, such reproductive impairments may have broader consequences for psychological well-being, quality of life, and social health, underscoring the importance of considering reproductive safety in the clinical management of patients receiving chronic topiramate therapy. Given the widespread use of this drug in individuals of reproductive age, further translational studies, particularly in humans, are urgently needed to clarify the dose- and time-dependency of these effects and to establish appropriate monitoring strategies for long-term users.

## Abbreviations

AMPA: α-amino-3-hydroxy-5-methyl-4-isoxazolepropionic acid
ANOVA: one-way analysis of variance
CA: carbonic anhydrase
CCEC/CCECs: cumulative concentration–effect curves
CNS: central nervous system (CNS)-acting drugs
DBF: Department of Biophysics and Pharmacology
*E*_max_: maximal effect, indicative of efficacy
GABA: γ-aminobutyric acid
LA: luminal area
–log EC_50_: negative logarithm of the concentration producing 50% of the maximal effect
MD: mean nuclear diameter
PBS: phosphate-buffered saline
PL/L: preleptotene and leptotene
SEC: seminiferous epithelium cycle
SEH: seminiferous epithelium height
SEM: standard error of the mean
STA: seminiferous tubule area
STD: seminiferous tubular diameter
